# Complete mitochondrial DNA genomes for two northeast Pacific mesopelagic fishes, the Mexican lampfish (*Triphoturus mexicanus*) and black-belly dragonfish (*Stomias atriventer*)

**DOI:** 10.1080/23802359.2017.1413293

**Published:** 2017-12-16

**Authors:** Andres Aguilar, Benson R. Truong, Frank Gutierrez

**Affiliations:** Department of Biological Sciences, California State University, Los Angeles, CA, USA

**Keywords:** Myctophidae, stomiidae, mesopelagic, mitogenome, gene rearrangements

## Abstract

Mesopelagic fishes are an important component of marine ecosystems, providing an important link between lower and higher trophic levels. This group of fishes is also highly abundant and make up a large portion of the marine vertebrate biomass. Here we report on the full mitochondrial sequences for two common mesopelagic fishes from the southern California bight: the Mexican lampfish *Triphoturus mexicanus* (Actinopterygii: Myctophidae) and the black-belly dragonfish *Stomias atriventer* (Actinopterygii: Stomiidae). *Triphoturus mexicanus* showed previously reported gene rearrangements for the Myctophidae. Phylogenetic analysis grouped *S. atriventer* with other Stomiiformes and *T. mexicanus* within the Myctophiformes.

Mesopelagic fishes make up the largest portion of the global vertebrate biomass (Gjøsaeter and Kawaguchi 1980; Kaartvedt et al. 2012; Davison et al. 2015). These fishes also make up an important component of marine food webs, as they are a link between plankton and higher-level predators (Gjøsaeter and Kawaguchi 1980; Cherel et al. 2008; Choy et al. 2012). Concordantly there is concern how this abundant and important fish community will respond to the impacts of climate change (Koslow et al. 2011; Asch 2015). Despite these features, there has been little genetic/genomic study on the mesopelagic fishes of the northeast Pacific.

We assembled the complete mitochondrial genomes for two common mesopelagic fishes from the southern California bight: the Mexican lampfish (*Triphoturus mexicanus*) and the black-belly dragonfish (*Stomias atriventer*). Samples were obtained from an Isaacs-Kidd midwater trawl in the San Pedro Channel (33.55830 N, −118.42550 W) and specimens, tissue and DNA are vouchered at CSULA (#16-001 and #16-033). Total genomic DNA was extracted and each individual was sequenced on an Illumina HiSeqX (Illumina, San Diego, CA). A portion of the reads from each species were assembled using NOVOPlasty (Dierckxsens et al. 2017) with a portion of the mitochondrial cytochrome oxidase I gene used as the initial seed. Resulting assemblies were annotated with on the MitoFish website (Iwasaki et al. 2013) and protein coding genes were used in a partitioned Bayesian phylogenetic analysis. PartitionFinder2 (Lanfear et al. 2016) was used to identify partitioning schemes and best-fit models of molecular evolution for the protein sequences.

The complete mitochondrial genome of *T. mexicanus* (Genbank accession no. MG321595) is 18,012 bp with two ribosomal genes (12S and 16S), 22 tRNAs and 13 protein-coding genes. It contained similar gene counts and organization as other Myctophidae and Lampanyctini (Poulsen et al. 2013). This includes the WANYC gene order, shifted tRNA-Cys and Tyr positions and a longer O_L_ region (90 bp in *T. mexicanus*). Specific characteristics of the Lampanyctini found in *T. mexicanus* include the relocation of the tRNA-Glu and intergenic non-coding regions resulting in the CytB/T/E/P gene order. The assembled *S. atriventer* mitogenome (Genbank accession no. MG321596) is 17,596 bp with two ribosomal genes (12S and 16S), 22 tRNAs and 13 protein-coding genes. *Stomias atriventer* contains the typical vertebrate gene order that is also observed in another Stomiiformes (*Diplophos* sp. – Miya and Nishida 2000).

Partitioned Bayesian phylogenetic analysis placed *T. mexicanus* sister to *T. nigrescens* with high posterior probability (Figure 1). *S. atriventer* placed in the Stomiioformes clade, sister to *C. sloani* +* S. gracilis* (Figure 1). This result is interesting given *S. gracilis* and *Diplophos* sp. are thought to be in the same family (Gonostomatidae), however a paraphyletic Gonostomatidae was found in a previous mitogenomic analysis (Miya et al. 2001) and the monophyly of the Stomiidae has been challenged with a mitochondrial and nuclear analysis of the family (Kenaley et al. 2014).

**Figure 1.**
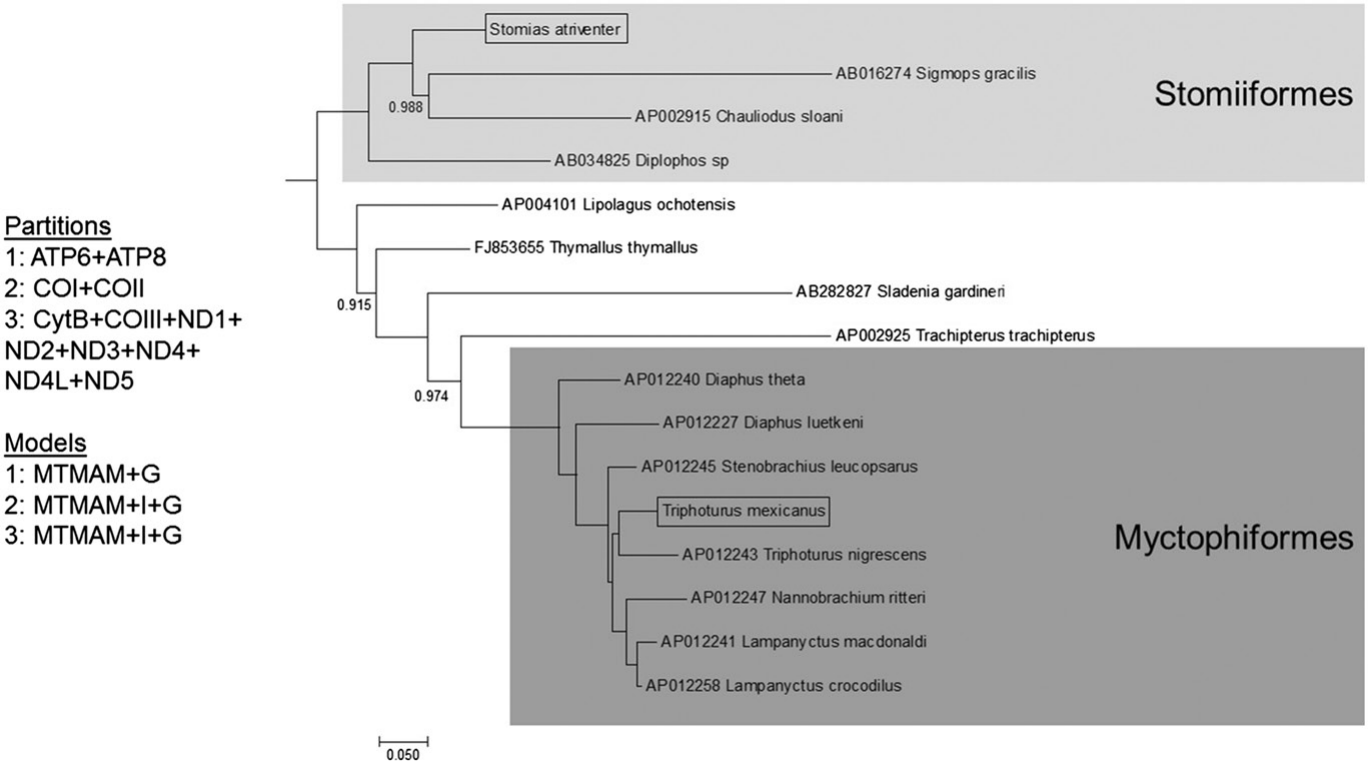
Partitioned Bayesian phylogenetic tree of 3623 amino acid positions for 16 teleost species, *T. mexicanus* and *S. atriventer* are in boxes. Bayesian analysis was run over two chains for 5 × 10^7^ generations, sampling each chain every 10^3^ generations with MrBayes (Ronquist and Huelsenbeck 2003). The first 25% of sampled trees were discarded as burning and only posterior probabilities below 1.0 are shown. Genes and models of evolution for each partition are also reported. The MTMAM model of protein evolution (Cao et al. 1998; Yang et al. 1998) with gamma or gamma plus invariant sites was the best model for each respective partition based on BIC in PartitionFinder2. GenBank accession numbers are shown for additional species included in this analysis.
